# miR-539 activates the SAPK/JNK signaling pathway to promote ferropotosis in colorectal cancer by directly targeting TIPE

**DOI:** 10.1038/s41420-021-00659-x

**Published:** 2021-10-02

**Authors:** Yan Yang, Zeyang Lin, Zhaopu Han, Zhengxin Wu, Jianyu Hua, Rui Zhong, Ruidan Zhao, Honggang Ran, Kaiyong Qu, Hongfei Huang, Huamei Tang, Jiyi Huang, Zhongchen Liu, Xuehui Hong, Zhihai Peng, Guohong Zhuang

**Affiliations:** 1 Xiamen Key Laboratory of Regeneration Medicine, Fujian Provincial Key Laboratory of Organ and Tissue Regeneration, Organ Transplantation Institute, School of Medicine, Xiamen University, 4221-122 Xiang An South Road, 361102 Xiamen, China; 2 Department of Pathology, Zhongshan Hospital Affiliated to Xiamen University, 361001 Xiamen, China; 3 School of Medicine, Xiamen University, 361102 Xiamen, China; 4 School of Medicine, Guangxi University, Nanning, China; 5 Department of General Surgery, Xiang An Hospital of Xiamen University, School of Medicine, Xiamen University, 2000 Xiang An East Road, 361102 Xiamen, China; 6 The First Affiliated Hospital of Xiamen University, Xiamen, Fujian China; 7 The Fifth Hospital of Xiamen, Xiamen, Fujian China; 8 Department of General Surgery, Shanghai 10th People’ s Hospital Affiliated to Tongji University, Shanghai, China; 9 Department of Gastrointestinal Surgery, Zhongshan Hospital, Xiamen, China

**Keywords:** Biomarkers, Cancer

## Abstract

Colorectal cancer (CRC) is a common tumor that harms human health with a high recurrence rate. It has been reported that the expression of microRNA-539 (miR-539) is low in several types of cancer, including CRC. Tumor necrosis factor (TNF)-α-induced protein 8 (TNFAIP8/TIPE) is highly expressed in CRC and promotes the proliferation, migration and angiogenesis of CRC. However, the relationship between miR-539 and TIPE and the mechanisms by which they regulate the proliferation of CRC remain to be explored. We aimed to investigate the functions and mechanisms of miR-539 in CRC proliferation. Functionally, miR-539 can bind to and regulate the expression of TIPE, and miR-539 activates SAPK/JNK to downregulate the expression of glutathione peroxidase 4 (GPX4) and promote ferroptosis. Our data reveal the novel role of miR-539 in regulating ferroptosis in CRC via activation of the SAPK/JNK axis, providing new insight into the mechanism of abnormal proliferation in CRC and a novel potential therapeutic target for advanced CRC.

## Introduction

Colorectal cancer (CRC) is the third leading cause of cancer-related death worldwide. Thus, as a key tool in early detection, prognostication, survival, and predicting the treatment response, biomarkers can be divided into diagnostic, prognostic, or predictive categories and provide a personalized indicator for therapeutic effectiveness [1].

MicroRNAs (miRNAs) have emerged as common biomarkers in recent years. MiRNAs are 17-25-nucleotide, noncoding RNA molecules [2]. Many reports indicate that miRNAs are involved in the carcinogenesis and disease progression [3, 4]. We note that it has been reported that miR-539 plays an anticancer role in cancer [5–8]. In particular, it has also been reported that miR-539 can inhibit the proliferation of CRC cells [9–11]. However, the mechanism by which miR-539 inhibits proliferation in CRC remains unclear.

MiRNAs are implicated in gene regulation through imperfect or perfect binding to the 3ʹ-untranslated regions (UTRs) of their target genes, resulting in translation inhibition and/or corresponding transcript degradation [12]. In our previous studies, we found TIPE can promote CRC angiogenesis, invasion and metastasis [13, 14]. Thus, we suspect that miR-539 functions by binding to TIPE.

It is worth noting that ferroptosis plays an important role in cancer according to recent reports [15, 16]. Ferroptosis is different from apoptosis, necrosis, and autophagic cell death based on morphological, biochemical, and genetic criteria [17]. In ferroptosis, toxic lipid reactive oxygen species (Lipid-ROS) are deposited under conditions of high concentrations of ferroptosis, thus inducing cell death [18]. Recent research has shown that ferroptosis due to GPX4 inactivation and reactive oxygen species (ROS) production can promote cell death in CRC [19, 20].

Here, we demonstrated the relationship between miR-539 and TIPE as well as the mechanistic role of miR-539 in ferroptosis. Our results showed that miR-539 activates the SAPK/JNK signaling pathway in CRC by targeting TIPE and reduces the expression of GPX4 to promote ferroptosis.

## Results

### miR-539 inhibits CRC proliferation by promoting ferroptosis in CRC

**Fig. 1:**
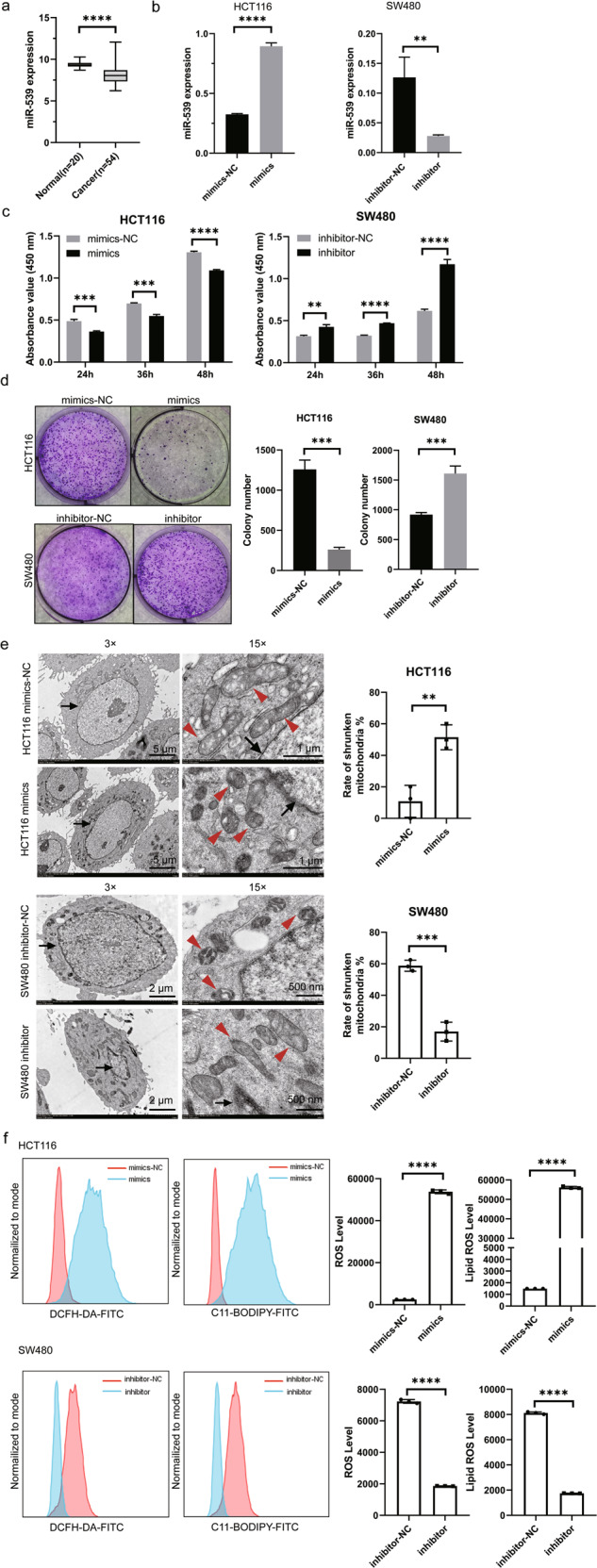
miR-539 inhibited CRC proliferation by promoting ferroptosis of CRC. **a** miR-539 expression in 20 normal tissues and 54 cancer tissues was analyzed based on GEO (GSE30454). HCT116 cells transfected with miR-539 mimic or miR-NC and the SW480 cells transfected with miR-539 inhibitor or inhibitor-NC for 24 h were collected, and the transfected cells were used in the subsequent assays. **b** The expression level of miR-539 was determined in HCT116 and SW480 cells after transfecting. **c** CCK-8 assays were conducted to examine the effects of miRNA-539 mimic and miR-539 inhibitor transfection on the proliferation of SW480 and HCT116 cells. **d** Representative colony formation assays were conducted to examine the effects of miRNA-539 mimic and miR-539 inhibitor transfection on the proliferation of SW480 and HCT116 cells. **e** The transfected cells were subjected to transmission electron microscopy and calculated the rate of shrunken mitochondria. Representative pictures are shown. Arrows, nuclei; arrowheads, mitochondria. **f** The transfected cells were labeled with ROS and Lipid ROS probes, the levels of ROS and Lipid ROS were detected by flow cytometry and analyzed statistically. Data are presented as the mean ± SD. **p < 0.01; ***p < 0.001; ****p < 0.0001.

To determine the role of miR-539 in CRC carcinogenesis, miR-539 expression in 20 colonic cancer mucosa and 54 normal colonic mucosa was analyzed in the GEO database (GSE30454), and it was downregulated in CRC cancer tissues (*p* < 0.0001, Fig. 1a). To further investigate the role of miR-539 in CRC, we transfected miR-539 mimics or miR-NC into HCT116 cells and miR-539 inhibitor or inhibitor-NC into SW480 cells (*p* < 0.05; *p* < 0.001, Fig. 1b). CCK-8 assays showed that the proliferation of HCT116 cells transfected with miR-539 mimics (HCT116-mimics) was significantly suppressed compared with that of HCT116 cells transfected with miR-NC (HCT116-mimics-NC) (*p* < 0.05), and the proliferation of SW480 cells transfected with miR-539 inhibitor (SW480-inhibitor) was significantly accelerated compared with that SW480 of cells transfected with inhibitor-NC (SW480-inhibitor-NC) (*p* < 0.05, Fig. 1c). Similarly, as shown in Fig. 1d, HCT116-mimics significantly decreased the number and size of surviving colonies (*p* < 0.05), and SW480-inhibitor increased those (*p* < 0.05). Together, these results indicate that miR-539 plays a tumor-suppressing role in CRC progression. Next, we aimed to determine the mechanism by which miR-539 affects CRC cell proliferation. Particularly, by transmission electron microscopy of the aforementioned cells, we observed shrunken mitochondria with increased membrane density (typical changes in ferroptosis) in HCT116-mimics and SW480-inhibitor-NC (Fig. 1e). The analysis of ROS and lipid ROS showed that compared with those transfected with the NC, the levels of ROS and lipid ROS in HCT116-mimics were significantly increased, and in SW480-inhibitor were decreased (*p* < 0.001, Fig. 1f). The above results indicate that miR-539 inhibits CRC proliferation by promoting ferroptosis in CRC.

### miR-539 interacts with TIPE

**Fig. 2:**
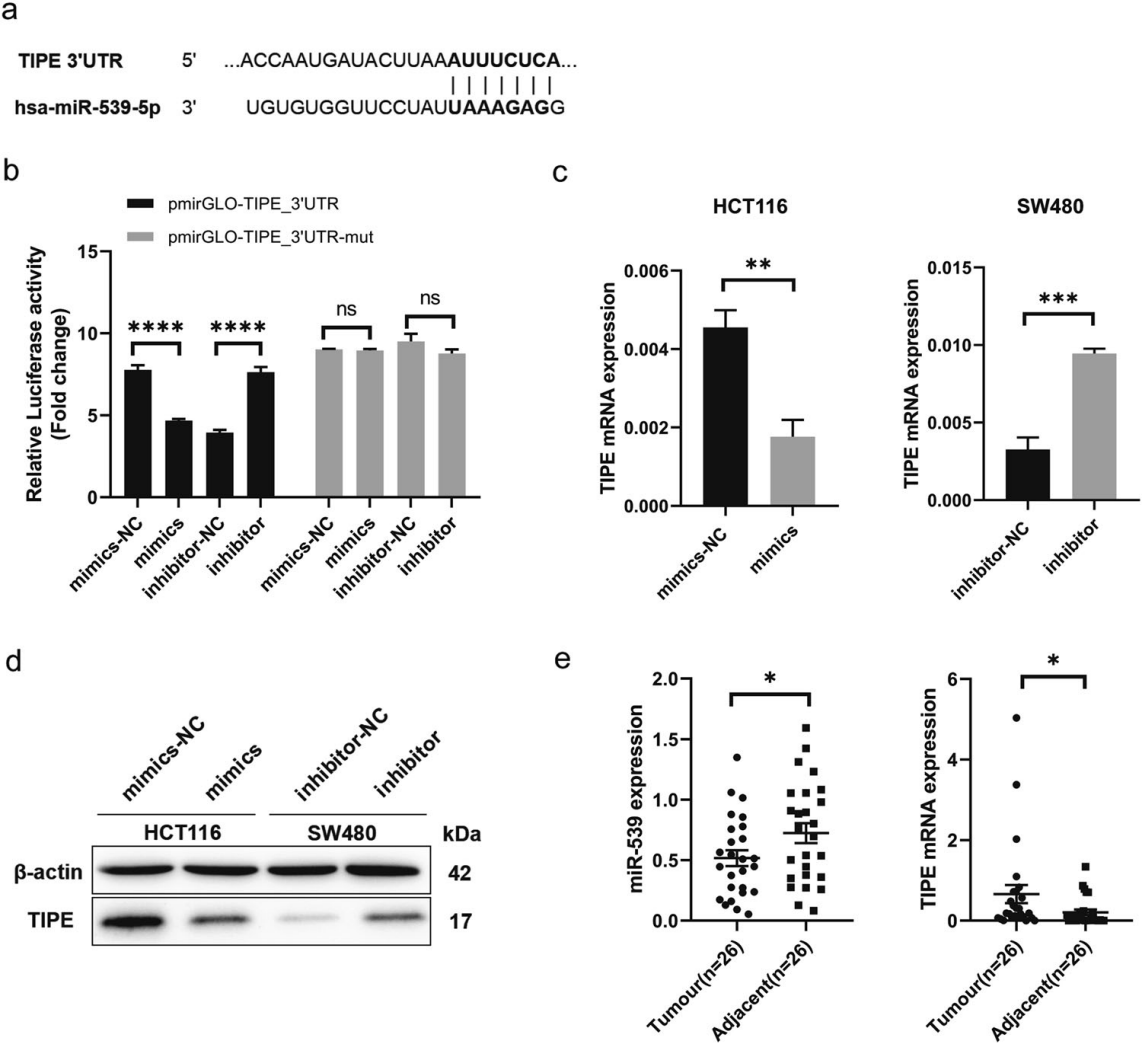
miR-539 interacted with TIPE. **a** Putative wild-type (Wt) miR-539 binding sites in the 3ʹ-UTR of TIPE. **b** Relative luciferase activities were analyzed in 293 T cells cotransfected with Wt or Mut reporter plasmids and miR-539 mimic or miR-NC and miR-539 inhibitor or inhibitor-NC. **c** Expression level of TIPE in HCT116 cell lines after the transfection with miRNA-539 mimic or miR-NC and SW480 cells transfected with miR-539 inhibitor or inhibitor-NC was analyzed by RT-qPCR. **d** Expression level of TIPE in HCT116 cell lines after the transfection with miRNA-539 mimic or miR-NC and SW480 cells transfected with miR-539 inhibitor or inhibitor-NC was analyzed by western blot. **e** miR-539 and TIPE expression in 26 pairs of CRC tissues and the corresponding adjacent normal tissues were analyzed by RT-qPCR. Data are presented as the mean ± SD. *p < 0.05; **p < 0.01; ***p < 0.001; ****p < 0.0001; ns no significant difference.

MiR-539 is expressed at low levels in CRC, while TIPE is expressed at high levels in CRC [14]. Therefore, we wanted to determine whether miR-539 regulates the transcription of TIPE. We first obtained sequence information of all small RNAs in a sample cell in a single sequence by high-throughput sequencing technology after transfection with TIPE shRNA in 293 T cells and found miR-539 was overexpressed in TIPE-knockdown cells (Supplementary Fig. S1). We used the public algorithm TargetScan 7.0 software (http://www.targetscan.org/) and predicted the TIPE 3ʹ-UTR with miR-539 binding sites (Fig. 2a). Then, luciferase reporter assays were performed on 293 T cells cotransfected with miR-539 mimics or mimics-NC and pGL3-TIPE-3ʹ-UTR or pGL3-TIPE-3ʹ-UTR Mut or miR-539 inhibitor or inhibitor-NC and pGL3-TIPE-3ʹ-UTR or pGL3-TIPE-3ʹ-UTR Mut to confirm this hypothesis. Restoring the expression of miR-539 significantly decreased the luciferase activities of the luciferase plasmid harboring the Wt binding sites, whereas mutation of the miR-539 binding site blocked this suppressive effect (Fig. 2b). Next, we confirmed that miR-539 can regulate TIPE expression. After transfection for 24 h, the expression of TIPE was reduced in HCT116-mimics and increased in SW480-inhibitor (*p* < 0.05; *p* < 0.001 Fig. 2c, d). In addition, miR-539 expression was significantly decreased in 26 CRC tissues compared with adjacent normal tissues but TIPE expression was upregulated in CRC tissues (*p* < 0.05, Fig. 2e). In summary, these results indicate that TIPE is a direct target of miR-539 in CRC cells and regulated by miR-539.

### miR-539 promotes ferroptosis by targeting TIPE in CRC

**Fig. 3:**
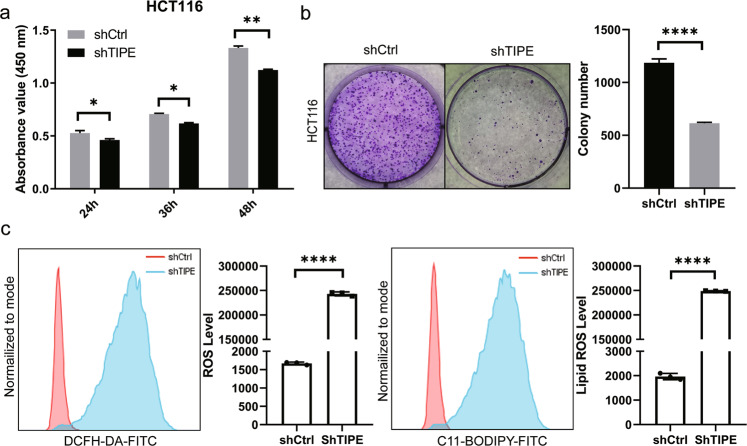
miR-539 promoted ferroptosis by targeting TIPE in CRC. **a**, **b** CCK-8 and representative colony formation assays were conducted to examine the effects of TIPE knockdown on the proliferation and colony formation of HCT116 cells. *p < 0.05; **p < 0.01; ****p < 0.0001. **c** The shCtrl and shTIPE HCT116 cells were labeled with ROS and Lipid ROS probes, the levels of ROS and Lipid ROS were detected by flow cytometry and analyzed statistically. Data are presented as the mean ± SD. *p < 0.05; **p < 0.01; ****p < 0.0001.

As described above, TIPE is a direct target of miR-539 in CRC, so we hypothesized that the tumor-suppressing roles of miR-539 in CRC cells could be imitated by TIPE knockdown. To confirm this hypothesis, we silenced TIPE expression in HCT116 cells by transfection with TIPE shRNA to establish shTIPE HCT116 cells (Supplementary Fig. S2a). Similar to miR-539 restoration, TIPE knockdown decreased the proliferation (*p* < 0.05, Fig. 3a) and colony formation (*p* < 0.05, Fig. 3b) of HCT116 cells. In addition, compared with those in shCtrl cells, the levels of ROS and lipid ROS in shTIPE HCT116 cells were significantly increased (*p* < 0.0001, Fig. 3c). These results further indicate that TIPE is a direct functional target of miR-539 in CRC and that miR-539 promotes ferroptosis by targeting TIPE in CRC.

### miR-539 regulates the expression of GPX4 through TIPE

**Fig. 4:**
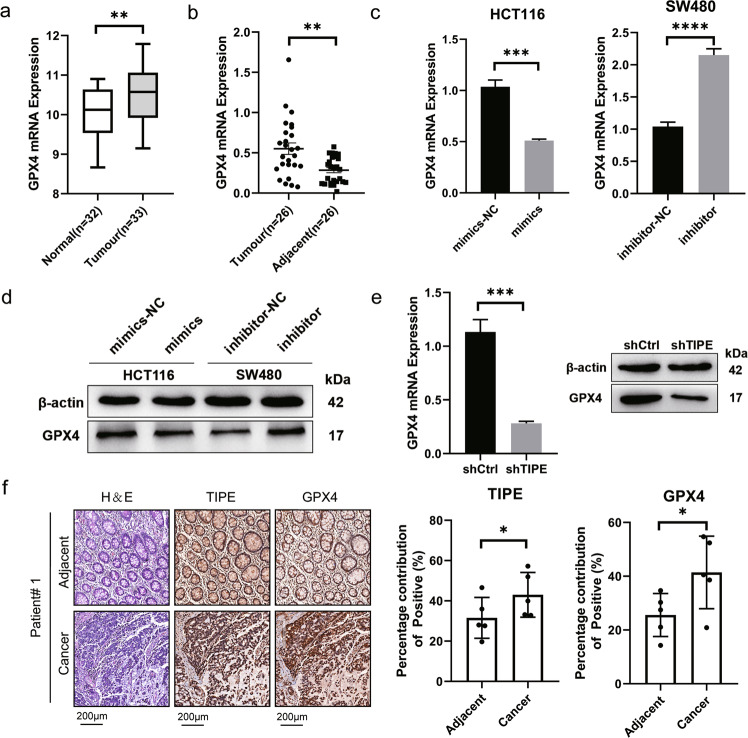
miR-539 regulates the expression of GPX4 through TIPE. **a** GPX4 expression in 32 normal tissues and 33 tumor tissues was analyzed based on GEO (GSE146009). **p < 0.01. **b** GPX4 expression in 26 pairs of CRC tissues and the corresponding adjacent normal tissues was analyzed by qRT-qPCR. **p < 0.01. **c**, **d** Expression level of GPX4 in HCT116 cell lines after the transfection with miRNA-539 mimic or miR-NC and SW480 cells transfected with miR-539 inhibitor or inhibitor-NC was analyzed by qRT-qPCR and western blot. **e** Expression level of GPX4 in shCtrl and shTIPE HCT116 cells was analyzed by RT-qPCR and western blot. **f** Representative images of TIPE and GPX4 expression in CRC tumor tissues and normal tissues were analyzed by IHC and the percentage contribution of positive was calculated. Data are presented as the mean ± SD. *p < 0.05; **p < 0.01; ***p < 0.001; ****p < 0.0001.

GPX4 is a major regulator of ferroptosis, a form of programmed cell death induced by iron-dependent lipid peroxidation. The loss of GPX4 can cause ferroptosis. Therefore, the expression of GPX4 in 32 normal tissues and 33 mucous adenocarcinoma tissues was analyzed in the GEO database (GSE146009). The results showed that GPX4 expression was upregulated in tumor tissues (*p* < 0.01, Fig. 4a), and GPX4 expression was upregulated in 26 CRC tissues compared with adjacent normal tissues (*p* < 0.01, Fig. 4b). Then, the results of RT-qPCR and western blotting showed that the expression of GPX4 was downregulated in HCT116-mimics and upregulated in SW480-inhibitor (*p* < 0.05, Fig. 4c, d). Moreover, the expression of GPX4 was downregulated in shTIPE HCT116 cells (*p* < 0.05, Fig. 4e). To investigate the relationship between TIPE and GPX4 expression and their clinical value, we evaluated their expression at the protein level with immunohistochemistry in the adjacent and cancer tissues of five patients. The results showed that in clinical samples, TIPE and GPX4 were expressed at higher levels in cancer tissues than in adjacent tissues (*p* < 0.05, Fig. 4f). The above results indicate that miR-539 regulates the expression of GPX4 through TIPE.

### miR-539 regulates GPX4 by promoting the activation of SAPK/JNK by targeting TIPE

**Fig. 5:**
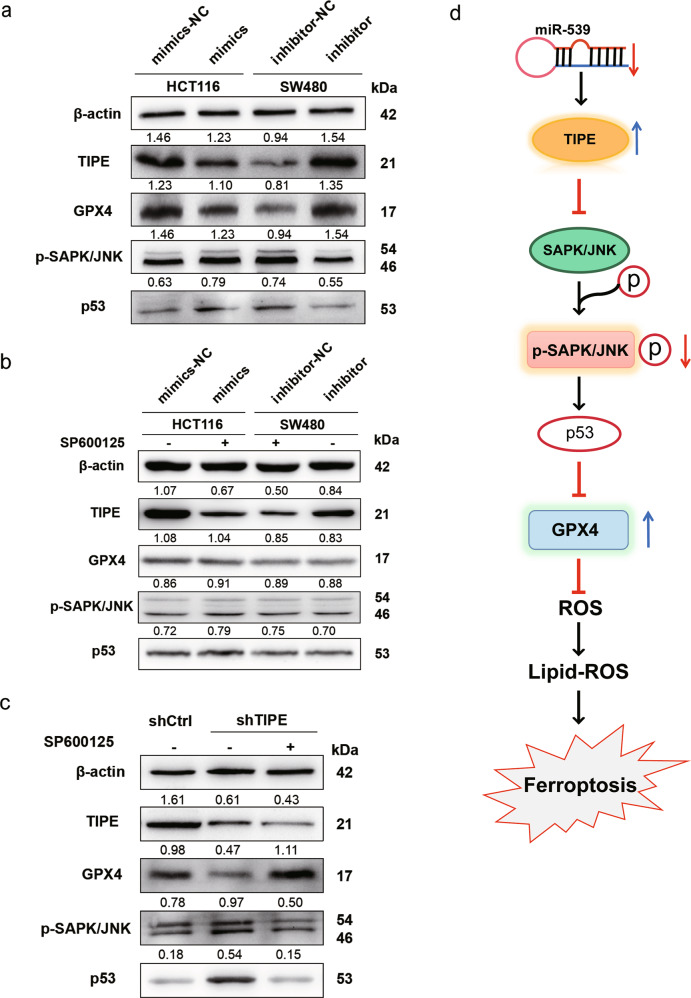
miR-539 regulates GPX4 through promoting the activation of SAPK/JNK by targeting TIPE. **a** HCT116 and SW480 cells were transfected with miR-539 mimic or miR-NC and miR-539 inhibitor or inhibitor-NC. After transfection for 24 h, the expression levels of TIPE, GPX4, p-SAPK/JNK and p53 were measured with western blot analysis. **b** HCT116 and SW480 cells were transfected with miR-539 mimic or miR-NC and miR-539 inhibitor or inhibitor-NC for 24 h, treat miR-NC and miR-539 inhibitor cells with inhibitor of JNK SP600125 (20 μM), the expression levels of TIPE, GPX4, p-SAPK/JNK and p53 were measured with western blot analysis. **c** The expression levels of TIPE, GPX4, p-SAPK/JNK and p53 were measured with western blot analysis in shCtrl, shTIPE and shTIPE treated with SP600125 (20 μM) for 24 h HCT116 cells. The value displayed above the WB result is the OD value after quantitative analysis. **d** Schematic diagram representing that downregulated miR-539 regulates GPX4 through inhibiting the activation of SAPK/JNK in CRC by targeting TIPE, afterwards, to inhibit ferroptosis and promote proliferation.

It has been reported that the expression level of p53 is closely related to the occurrence of ferroptosis. In addition, SAPK/JNK is the upstream molecule of p53. To explore whether miR-539 contributes to the regulation of the SAPK/JNK pathway by targeting TIPE and further affects the expression of GPX4 in CRC, western blot analysis was performed to detect the expression levels of phospho-SAPK/JNK (p- SAPK/JNK) and p53. The results showed that in HCT116-mimics and SW480-inhibitor-NC, the expression of p-SAPK/JNK and p53 was upregulated, while the expression of TIPE and GPX4 was downregulated (Fig. 5a). Then, we treated HCT116-mimics and SW480-inhibitor-NC with an inhibitor of JNK (SP600125 20 µM). As shown in Fig. 5b, SP600125 restored GPX4 expression in HCT116-mimics and SW480-inhibitor-NC by inhibiting the SAPK/JNK pathway but the expression of TIPE was not affected. Moreover, the expression levels of these proteins were analyzed in shCtrl, shTIPE and shTIPE HCT116 cells treated with SP600125. Similarly, as shown in Fig. 5c, compared with shCtrl HCT116 cells, the expression of GPX4 and TIPE was decreased in shTIPE HCT116 cells, and the expression of p-SAPK/JNK and p53 was increased. After treatment with SP600125, compared with shTIPE cells, the expression of GPX4 was restored while the p-SAPK/JNK and p53 were inhibited, and the expression of TIPE was not affected by SP600125. These results indicate that miR-539 activates the SAPK/JNK signaling pathway in CRC by targeting TIPE and reduces the expression of GPX4 to promotes ferroptosis (Fig. 5d).

### Upregulated TIPE promotes CRC tumor growth in vivo by inhibiting GPX4-induced ferroptosis

**Fig. 6:**
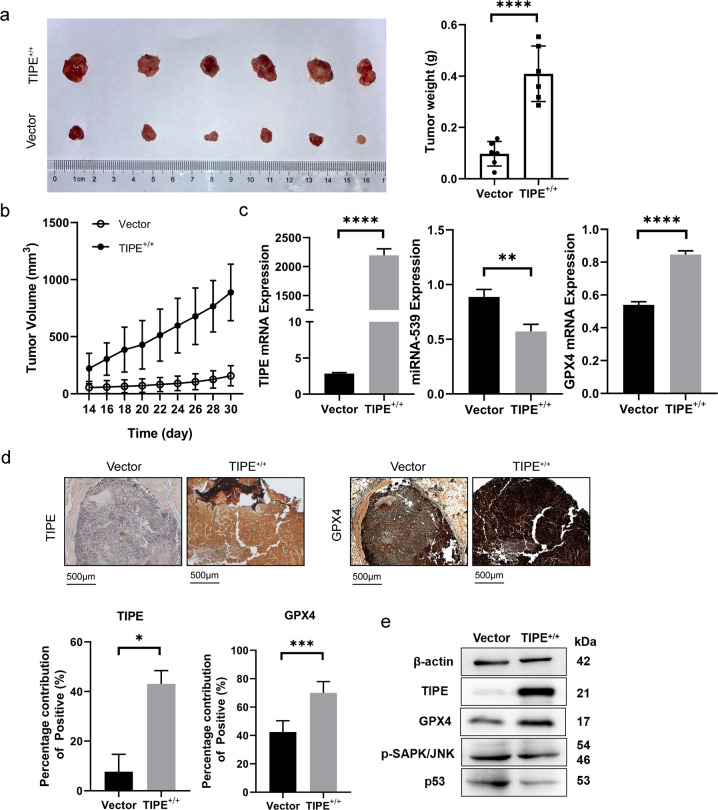
Upregulated TIPE promotes CRC tumor growth in vivo by GPX4-induced ferroptosis inhibition. **a** Representative images of the TIPE^+/+^ and vector groups xenograft tumors and the weights of the xenograft tumors from the TIPE^+/+^ and vector groups. **b** The volume of the xenograft tumor from the TIPE^+/+^ and vector groups was calculated every 2 days after inoculation for 2 weeks. *p* < 0.0001. **c** TIPE, miR-539 and GPX4 expression in the TIPE^+/+^ and vector groups was analyzed by RT-qPCR. **d** Representative images of the TIPE and GPX4 expression in the TIPE^+/+^ and vector groups was analyzed by IHC. **e** The expression levels of TIPE, GPX4, p-SAPK/JNK and p53 in the TIPE^+/+^ and vector groups were measured with western blot analysis. Data are presented as the mean ± SD. **p < 0.01; ****p < 0.0001.

Our previous studies confirmed that miR-539 can regulate TIPE. To investigate the effect of miR-539 on tumor growth in vivo, we established a stable TIPE^+^^/^^+^ SW480 cell line by transfection with a TIPE overexpression plasmid (Supplementary Fig. S2b). TIPE^+/+^ SW480 cells and control cells were subcutaneously inoculated into nude mice. Nude mice were sacrificed on day 30 following inoculation, and xenografts were excised and weighed. The tumor volume and weight of the TIPE^+/+^ SW480 cell group were significantly higher than those of the control cell group (*p* < 0.001, Fig. 6a, b). Furthermore, RT-qPCR analysis indicated that the expression level of miR-539 was significantly lower in TIPE^+/+^ SW480 cells than in control cells, and the expression levels of TIPE and GPX4 were significantly higher in TIPE^+/+^ SW480 cells (*p* < 0.05, Fig. 6c). To investigate the relationship between TIPE and GPX4 expression and their clinical value, we evaluated their expression at the protein level with immunohistochemistry and we found that TIPE and GPX4 expression was positive in TIPE^+/+^ SW480 cells (Fig. 6d). Western blot analysis indicated that p-SAPK/JNK and p53 were significantly decreased in TIPE^+/+^ SW480 cells and GPX4 was increased (Fig. 6e). These results indicate that upregulated TIPE promotes CRC tumor growth in vivo by inhibiting ferroptosis through the SAPK/JNK signaling pathway.

## Discussion

Increasing evidence has shown that a large number of miRNAs are involved in the formation and progression of CRC [21]. Thus, an miRNA-based targeted therapy that inhibits or restores expression may provide a promising therapeutic approach for anticancer therapy [12].

In our study, we studied the expression of miR-539 in CRC tissues and normal tissues and found that miR-539 is expressed at low levels in CRC tissues. According to reports, miR-539 is also expressed at low levels in liver cancer, non-small cell lung cancer, breast cancer, and pancreatic cancer, as it inhibits cell proliferation, migration, and invasion and EMT to inhibit tumor growth [5–8, 22]. These findings indicate that miR-539 has tumor-suppressive effects in human cancers and may be a new and effective noninvasive target for the diagnosis of patients with these specific tumor types. Thus, we hypothesized that miR-539 is a tumor suppressor in CRC.

Functional experiments indicated that exogenous miR-539 expression inhibited CRC cell proliferation. Generally, apoptosis is the main pathway that affects the proliferation of cancer cells, and it has been reported that miRNAs can affect the proliferation of CRC cells through the apoptosis pathway [23, 24]. However, we did not find that exogenous miR-539 expression affected the apoptosis rate of CRC cells (Supplementary Fig. S3a), nor did we observe typical apoptotic bodies in the CRC cell lines under an electron microscope. Following the early stage of apoptosis, a phenomenon known as karyorrhexis, which refers to the fragmentation of the nucleus, ensues. Karyorrhexis is accompanied by further blebbing of the plasma membrane that tears apoptotic cells into small apoptotic bodies [25]. Thus, we focused on ferroptosis, another method of cell death. Ferroptosis is a nonapoptotic form of cell death that is mainly caused by a decrease in glutathione (GSH) synthesis and decreased activity of GPX4 [26, 27]. Consistent with our hypothesis, our results confirmed that exogenous miR-539 expression can induce ferroptosis in vitro and decrease tumor growth in vivo. Specifically, we observed the effect of miR-539 on the morphology of CRC cells, which exhibit smaller than normal mitochondria with increased membrane density and collapsed mitochondrial cristae [15]. Then, we found that exogenous miR-539 downregulated GPX4 expression. This result further led to the accumulation of ROS and toxic L-ROS and caused ferroptosis.

Many miRNAs are dysregulated in cancer tissue compared with normal tissue [28–30], acting either as tumor suppressors or oncogenes depending on the functions of their target genes [31, 32]. Interestingly, we found that TIPE is highly expressed in CRC tissues by bioinformatics and qRT-PCR analyses. Based on our previous research, we hypothesized that TIPE, as an important molecule in the development and progression of CRC, could be a target molecule of miR-539 [13, 14, 33]. Then, as expected, we demonstrated that TIPE was a novel target gene of miR-539 in CRC and that miR-539 promotes ferroptosis by binding to TIPE and inhibiting its expression; additionally, TIPE had no effect on apoptosis (Supplementary Fig. S3b). We further confirmed our idea in vivo that overexpression of TIPE can promote tumor growth in vivo by inhibiting ferroptosis.

Moreover, we demonstrated that miR-539 induced ferroptosis in CRC cells by targeting TIPE through the SAPK/JNK signaling pathway. Although it is commonly accepted that p53-mediated cell cycle arrest, apoptosis, and senescence all serve as major mechanisms of tumor suppression, accumulating evidence indicates that other activities of p53, such as metabolic regulation, are also critical for tumor suppression [34, 35]. It was previously reported that in tumor cells, ferroptosis is believed to be an endogenous anticancer mechanism downstream of the tumor suppressor gene p53 [36, 37]. p53 has been found to participate in this pathway, inhibiting the function of System Xc-, an anti-transporter of cystine/glutamate, by downregulating the expression of SLC7A11 [15, 36]. In our study, we examined the effect of miR-539 on the phosphorylation levels of p53 and its upstream molecule SAPK/JNK, and our conclusions further support these findings. However, the specific mechanism by which TIPE inhibits the SAPK/JNK pathway in CRC still needs to be studied.

In conclusion, this study showed that miR-539 is significantly downregulated in CRC tissues and cell lines. This miRNA can potently inhibit CRC cell proliferation, increase cell ferroptosis in vitro and reduce tumor growth in vivo by directly targeting TIPE and activating the SAPK/JNK signaling pathway. These results suggest that miR-539 is a new therapeutic target for CRC patients.

## Materials and methods

### Tissue samples

Tumor and paired adjacent tissues from 26 patients with CRC who had not received radiotherapy and chemotherapy prior to surgery were collected from Zhongshan Hospital Affiliated with Xiamen University (Xiamen, China).

### Cell culture

HCT116 and SW480 and the 293T cell line were obtained from the Anti-Cancer Center of Xiamen University (Xiamen, Fujian). HCT116 and 293T cells were cultured in Dulbecco’s modified Eagle’s medium (DMEM, Gibco, CA, USA) and SW480 cells were cultured in Roswell Park Memorial Institute 1640 (RPMI-1640, Gibco, CA, USA) supplemented with 10% fetal bovine serum (FBS, Gibco), 100 units/ml penicillin and 100 mg/ml streptomycin (Invitrogen, Carlsbad, CA, USA). All cell lines were cultured in a humidified environment containing 5% CO_2_ at 37 °C.

### Transfection

MiR-539 mimic, miRNA mimic negative control (miR-NC), miR-539 inhibitor and miRNA inhibitor negative control (inhibitor-NC) were chemically produced by GenePharma Co., Ltd. (Shanghai, China). Transfection was performed using Lipofectamine® 2000 (Invitrogen, Carlsbad, CA, USA).

### Quantitative real-time PCR (qRT-PCR)

Total RNA was isolated from tissues and cells with TRIzol reagent according to the manufacturer’s instructions. One microgram of RNA was used as a template and reverse transcribed into cDNA with an All-in-one First-Stand cDNA Synthesis SuperMix for qPCR kit or a TransScript® Green miRNA Two-Step qRT-PCR SuperMix kit according to the manufacturer’s protocols. qRT-PCR was performed using an All-in-one First-Stand cDNA Synthesis SuperMix for qPCR kit or a TransScript® Green miRNA Two-Step qRT-PCR SuperMix kit, and data acquisition was enforced on an Applied Biosystems real-time PCR System (Life Technologies, Hercules, California, USA). The primer sequences (5ʹ → 3ʹ) were as follows:

hsa-miR-539-5p: CGCGGAGAAATTATCCTTGGTGTGT;

U6-F: CTCGCTTCGGCAGCACA;

U6-R: AACGCTTCACGAATTTGCGT;

TIPE-F: TTCAGGCCTCCCTCTTTAACAATC;

TIPE-R: CGTTCGTGGCAGGGGTTATT;

GPX4-F: ATGGTTAACCTGGACAAGTACC;

GPX4-R: GACGAGCTGAGTGTAGTTTACT;

ACTIN-F: AGCGAGCATCCCCCAAAGTT;

ACTIN-R: GGGCACGAAGGCTCATCATT.

The relative gene expression levels were determined using β-actin or U6 as the control with the following formula: Ratio (reference/target) = 2^CT(reference) – CT(target).^

All reagents are purchased at Transgen, Beijing, China.

### Cell counting kit-8 (CCK-8) and colony formation assays

The CCK-8 assay was utilized to determine cell proliferation. The transfected cells were collected at 24 h posttransfection and prepared as a single-cell solution. A total of 5 × 10^3^ cells were added to 96-well plates and cultured at 37 °C in a humidified incubator for 24, 36, and 48 h. At the end of culturing, use the CCK-8 kit (Biosharp, Inc., Labgic, China) to analyze as previously described [33].

The colony formation assay was used to determine colony formation. After transfection for 24 h, cells were harvested, resuspended, and plated into six-well plates at a density of 1000 cells per well. The cells were incubated at 37 °C in a humidified incubator for 14 days. On day 15, colony formation assays were performed, and surviving colonies (>50 cells per colony) were counted following fixation with 4% paraformaldehyde and staining with 0.1% crystal violet.

### Transmission electron microscopy

CRC cells were collected in sterile tubes and fixed with 3% glutaraldehyde solution overnight at 4 °C. Samples were then embedded in Spurr’s resin, followed by ultrathin sectioning. Images were taken with a Hitachi HT-7800 TEM (Tokyo, Japan). The quantification of intact or shrunken mitochondria was carried out for at least three different fields, and the percentage was calculated as the number of shrunken mitochondria divided by the total number of mitochondria in each field.

### Reactive oxygen species and lipid peroxidation assays

CRC cells were seeded into a 24-well plate. Cells were cultured in 200 μl serum-free medium containing 10 μM DCFH-DA (Yeasen) and 10 μM BODIPY-C11 (Thermo Fisher) respectively and incubated for 30 min at 37 °C in a cell culture incubator. Cells were washed and resuspended in 500 μL fresh phosphate-buffered saline (PBS) and then analyzed immediately with a Beckman cytometer (BD Biosciences). The ratio of mean fluorescence intensities was analyzed using FlowJo v X 10.0.7 software (Tree Star, Inc., Ashland, OR).

### Luciferase reporter assay

The 3ʹ-UTR of TIPE containing the wild-type (Wt) miR-539 putative binding sites and mutant (Mut) binding sites was designed and chemically synthesized by GenePharma Co., Ltd., and inserted into the pGL3 luciferase vector (Promega Corporation, Madison, WI, USA) to construct pGL3-TIPE-3ʹ-UTR Wt and pGL3-TIPE-3ʹ-UTR Mut, respectively. Cells were inoculated in triplicate in 24-well plates and allowed to settle for 24 h. Next, the cells were cotransfected with miR-539 mimics or miR-NC and miR-539 inhibitor or inhibitor-NC with pGL3-TIPE-3ʹ-UTR Wt or pGL3-TIPE-3ʹ- UTR Mut using Lipofectamine® 2000 and tests were performed after 48 h in accordance with the manufacturer’s protocol.

### Western blot analysis

The proteins were extracted as described previously [33], and equal amounts of cell lysates (20-40 µg of proteins) were subjected to 12% SDS-PAGE. After electrophoresis, followed by incubation with anti-TIPE (1:2000; Abcam, Cambridge, MA, USA), anti-GPX4 (1:10,000; Abcam, Cambridge, MA, USA), phospho-SAPK/JNK (1:1000; Cell Signaling), anti-p53 (1:1000; Cell Signaling), or anti-β-actin (1:1000; ZSGB-Bio, Beijing, China) antibodies at 4 °C overnight. Then, the membranes were rinsed and probed with horseradish peroxidase (HRP)-conjugated goat anti-rabbit IgG (1:10,000; ZSGB-Bio) for 1 h at room temperature. Then, the immunoreactive bands were detected on a Tanon 5200 Series Automatic Chemiluminescence/Fluorescence Image Analysis System (Tanon, Shanghai, China).

### Xenograft experiment

Six 4-week-old male BALB/c nude mice were ordered from the Shanghai Laboratory Animal Center (Shanghai SLAC Laboratory Animal Co., Ltd., China). A total of 5 × 10^6^ TIPE^+/+^ SW480 cells were suspended in 100 µL of PBS and subcutaneously injected into the right axilla flank of each nude mouse, and the same amount of vector SW480 cells was into the left. At 2 weeks after inoculation, the xenograft tumor size was measured using Vernier calipers every 2 days, and tumor volume was calculated according to the following formula: 1/2 × tumor length × tumor width. Mice were sacrificed 30 days after inoculation. Tumor xenografts were excised, weighed, and analyzed by immunohistochemistry, qRT-PCR, and western blot.

### Immunohistology

Tissues from CRC patients and tumor xenografts from nude mice were fixed in 10% neutral buffered formalin for 24 h and embedded in paraffin; 5-μm paraffin sections were stained with H&E.

Paraffin sections were subjected to gradient hydration and heat-induced antigen retrieval. Incubate the antibody as described previously. Visualization was induced with a diaminobenzidine substrate kit (Thermo Fisher). The following primary antibodies and reagents were used: TIPE, 1:200, ab; GPX4, 1:200, ab. Representative images from H&E and immunohistology sections were captured using a microdigital section scanning system (Motic VM1, China).

### Statistical analysis

The experimental data were analyzed with GraphPad Prism 8 software. We used the paired *t* test or unpaired *t* test to evaluate the significance of the difference between groups, and the data are presented as the mean ± SD from three separate experiments. *p* < 0.05 was used to indicate a statistically significant difference.

## Supplementary information

Supplementary information

Figure S1

Figure S2

Figure S3

## Data Availability

The datasets used and/or analyzed during the current study are available from the corresponding authors on reasonable request.
