# Supplementary material for: miR-539 activates the SAPK/JNK signaling pathway to promote ferropotosis in colorectal cancer by directly targeting TIPE
Source: Cell Death Discov. 2021 Oct 2;7:272. doi: 10.1038/s41420-021-00659-x (PMC8487425; doi:10.1038/s41420-021-00659-x)
Supplement: Supplementary file 1 — Supplementary information [file 41420_2021_659_MOESM1_ESM.docx]

**Supplementary Materials for**

**miR-539 suppresses the SAPK/JNK signaling pathway to inhibit ferropotosis in colorectal cancer by directly targeting TIPE**

Yan Yang, Zeyang Lin, Zhaopu Han, Zhengxin Wu, Jianyu Hua, Rui Zhong, Ruidan Zhao, Honggang Ran, Kaiyong Qu, Hongfei Huang, Huamei Tang, Jiyi Huang, Zhongchen Liu, Xuehui Hong, Zhihai Peng, Guohong Zhuang

Contact: Guohong Zhuang, Email: zhgh@xmu.edu.cn; Xuehui Hong, Email:hongxu@xmu.edu.cn; Zhihai Peng, Email:pengzhihai1958@163.com

**Supplementary information, Figures**

Figure S1**.** miR-539 was overexpressed in TIPE-knockdown cells

We obtained sequence information of all small RNAs in a sample cell in a single sequence by high-throughput sequencing technology after transfection with TIPE shRNA in 293T cells. We screened 10 of the most [up-regulated](D:/soft/youdao/Dict/8.9.6.0/resultui/html/index.html#/javascript:;) miRNAs in TIPE-knockdown cells and 10 of the most under-regulated miRNAs in control cells. Consistent with our hypothesis, miR-539 was overexpressed in TIPE-knockdown cells.

Figure S2. Construction of stable transfected cell lines

shRNA-mediated silencing (Figure S2a) and forced expression (Figure S2b) of TIPE. The efficiency of TIPE overexpression and TIPE knockdown was checked by Western blotting and qRT-PCR, respectively. ****p<0.0001, mean±S.D.m in three separate experiments.

Figure S3. miR-539 and TIPE expression didn’t affect the apoptosis rate of CRC cells.

The effect of miR-539 on CRC cell apoptosis was determined using flow cytometric analysis. In HCT116 and SW480 cells, apoptosis rates were not significantly different after transfection with miR-539 mimics or inhibitors compared with control cells (p=0.2880, p=0.2431, Figure S3a). Similarly, in shCtrl and shTIPE HCT116 cells, apoptosis rates were not significantly different (p=0.7446,Figure S3b).

**Supplementary information, Materials and Methods**

**Oligonucleotides, siRNAs, plasmids and transfection**

A lentiviral system was used to establish stably transfected cells. The lentiviral vector (pSIREN-RetroQ) was a gift from the laboratory of Professor Jin Guanghui, School of Medicine, XMU. The vector encoding the indicated interference fragment of TIPE was constructed and verified. A retroviral vector (PLNCX-2) containing TIPE cDNA was also constructed and tested. The TIPE or shTIPE plasmid and packaging vector were added to 293T cells according to the manufacturers' instructions (jet PRIME, Polyplus, New York, USA) to prepare retroviruses. The viral supernatant was harvested and centrifuged at 1000 rpm for 3 minutes to remove the cell debris. Forty-eight hours after transfection, CRC cells growing in the logarithmic phase were transferred into a 6-well plate (at 60% confluency). CRC cells stably overexpressing TIPE were selected by exposure to 600 mg/ml G418 (Thermo Fisher Scientific Inc.). Stable shTIPE cells were selected by exposure to 6 ng/ml puromycin (Sigma-Aldrich, St. Louis, MO, USA). These cells were cultured in continuous screening medium for seven days, and then the concentrations of the drugs were reduced (300 g of G418, 3 ng of puromycin) followed by an additional two weeks of incubation. The expression of TIPE in the indicated cells was confirmed by qPCR and Western blotting.

**Flow Cytometry**

The apoptosis of HCT116 and SW480 cells in different treatment groups was examined by flow cytometry. Cells were seeded into six-well plates at a density of 1×105 per well and incubated for 24 h. The cells were then harvested, washed twice with fluorescence-activated cell sorting (FACS) wash buffer (PBS containing 1% FBS), and 500 ml propidium iodide (PI) working solution (Meilunbio, Dalian,China) was added to each cell sample; the samples were gently mixed to completely resuspend the cell pellet and then incubated at 37°C for 30 min in the dark. After staining, a CytoFlex S flow cytometer (Beckman Coulter, CA, USA) was used to analyze cell fluorescence, and FlowJo software (Stanford University, USA) was used for analysis.
