# Supplementary figures and images for: miR-539 activates the SAPK/JNK signaling pathway to promote ferropotosis in colorectal cancer by directly targeting TIPE
Source: Cell Death Discov. 2021 Oct 2;7:272. doi: 10.1038/s41420-021-00659-x (PMC8487425; doi:10.1038/s41420-021-00659-x)

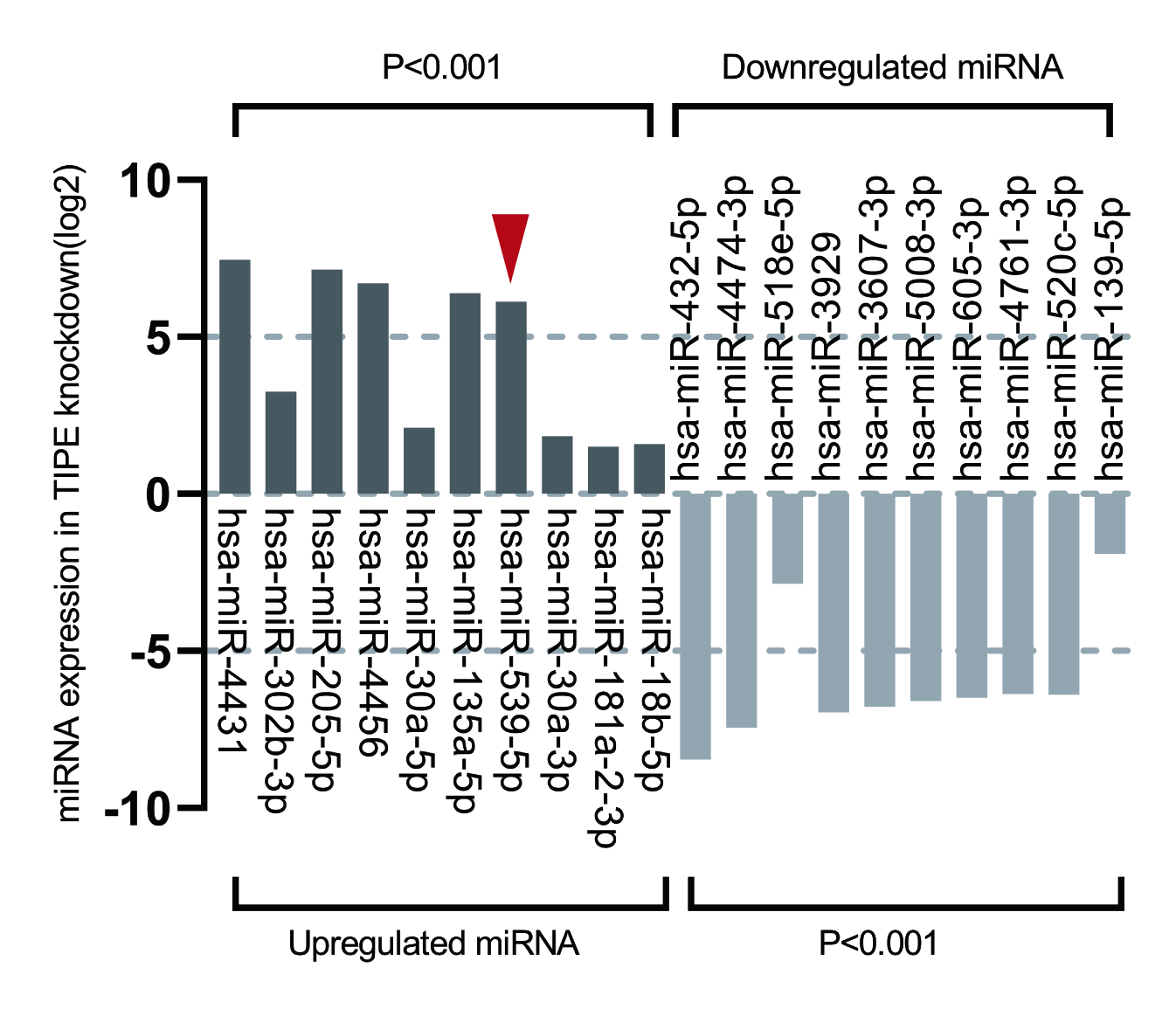

Supplement: Supplementary file 2 — Figure S1 [file 41420_2021_659_MOESM2_ESM.tif]

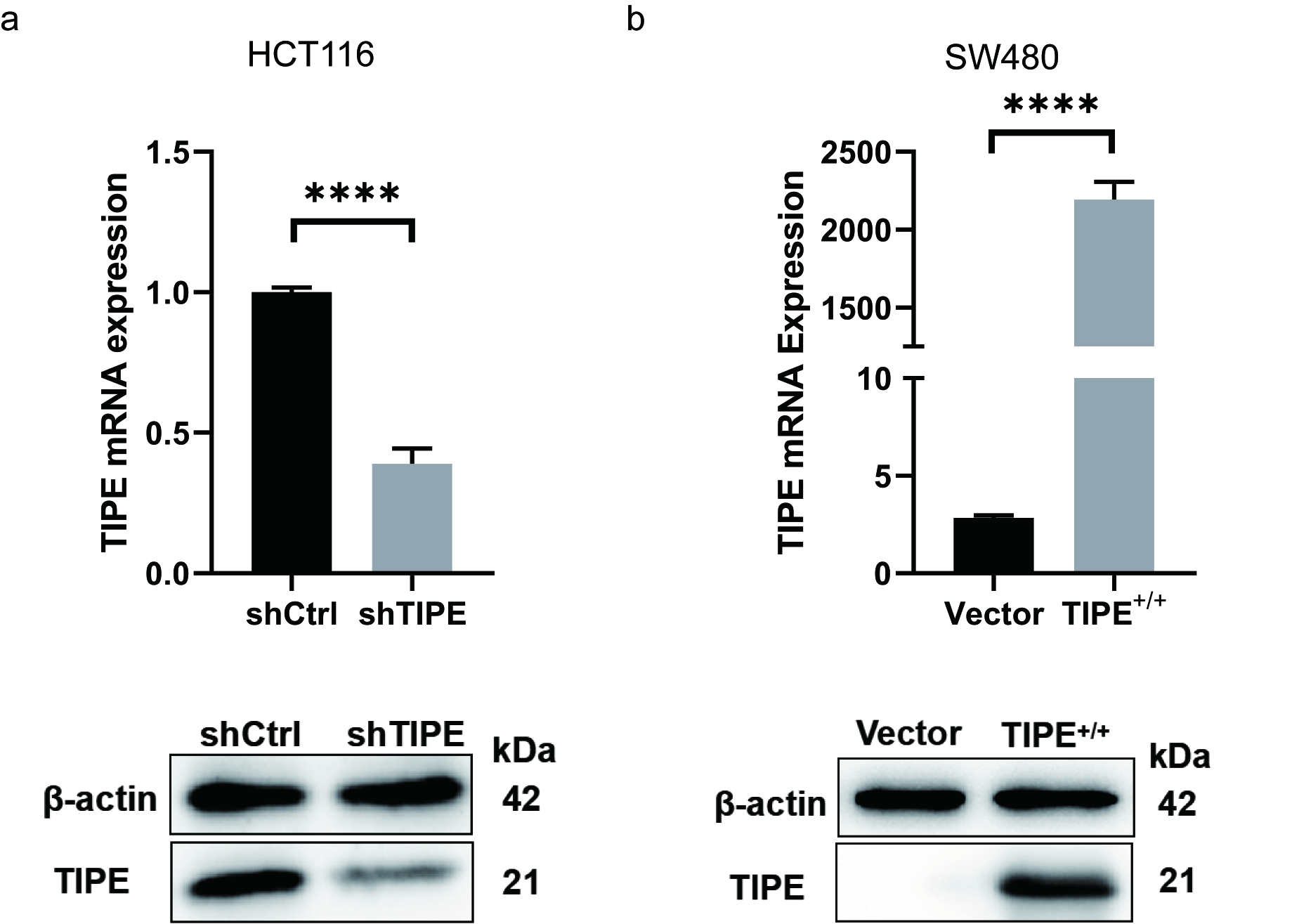

Supplement: Supplementary file 3 — Figure S2 [file 41420_2021_659_MOESM3_ESM.tif]

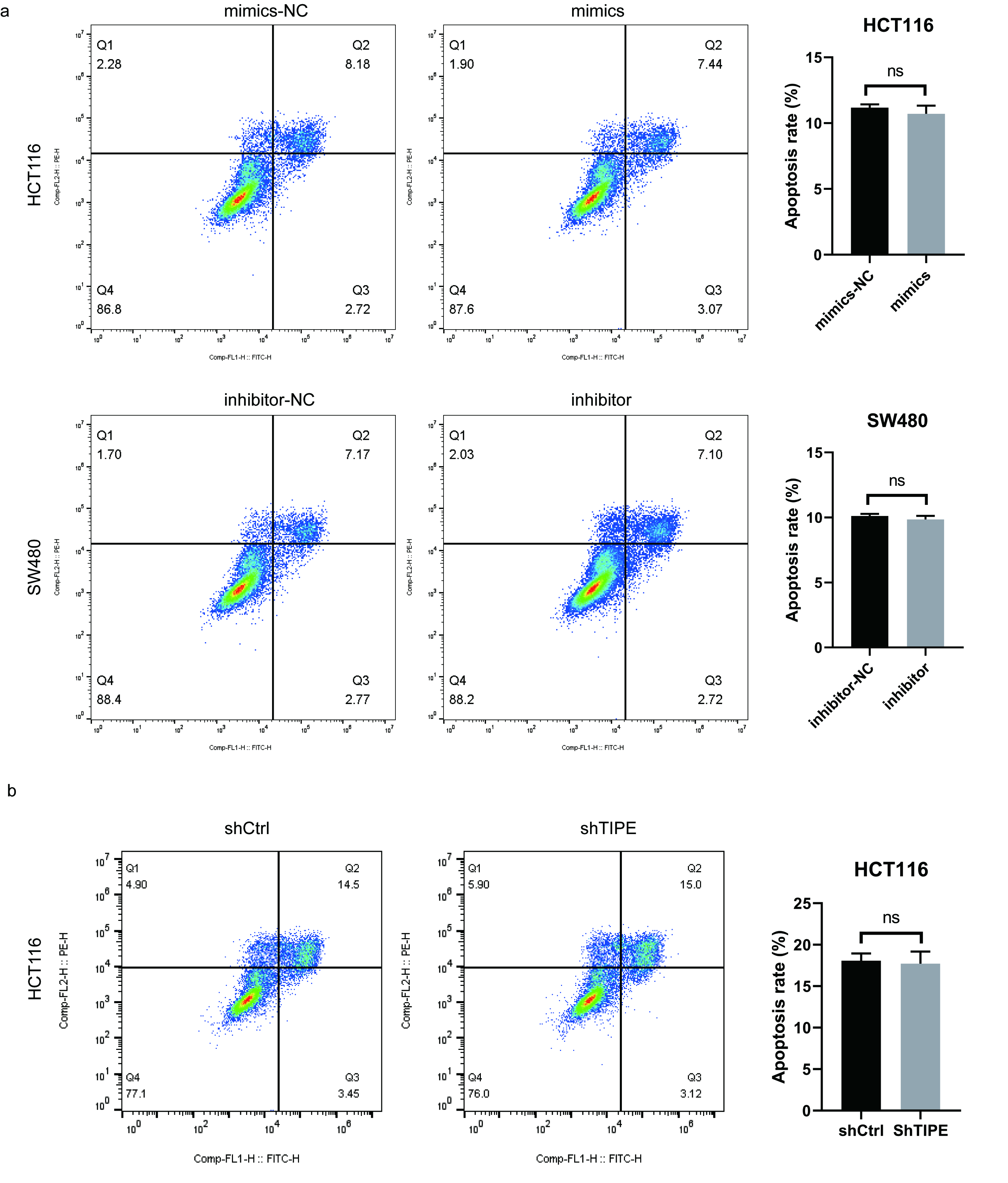

Supplement: Supplementary file 4 — Figure S3 [file 41420_2021_659_MOESM4_ESM.tif]
